# The comparison of the intensity of human intestinal spirochetes between *Brachyspira pilosicoli* and *Brachyspira aalborgi* infections

**DOI:** 10.3164/jcbn.18-68

**Published:** 2018-11-28

**Authors:** Junichi Iwamoto, Yoshikazu Adachi, Akira Honda, Tadakuni Monma, Yasushi Matsuzaki

**Affiliations:** 1 Department of Gastroenterology, Tokyo Medical University Ibaraki Medical Center, 3-20-1 Ami-machi Chuo, Inashiki-gun, Ibaraki 300-0395, Japan; 2 Animal Health Laboratory, School of Agriculture, Ibaraki University, 3-21-1 Ami, Ibaraki 300-0393, Japan; 3 Joint Research Center, Tokyo Medical University Ibaraki Medical Center, 3-20-1 Ami-machi Chuo, Inashiki-gun, Ibaraki 300-0395, Japan

**Keywords:** agglutination, antibody, titer, *Brachyspira*

## Abstract

The agglutination titers of *Brachyspira pilosicoli* (*B. pilosicoli*) and *Brachyspira aalborgi* (*B. aalborgi*) were examined in colitis patients with human intestinal spirochetes. Among three cases of colitis patients, the titer of *B. pilosicoli* was extremely high in two cases while the titer of *B. aalborgi* was extremely high in one case. These three cases had symptoms of colitis, such as watery diarrhea, and we diagnosed the case as *Brachyspira-* related colitis. These findings suggest that the agglutination titers of *Brachyspira* may be useful in cases of *Brachyspira-* related colitis. Severe symptoms, such as abdominal pain and diarrhea, were observed in cases with high antibody titer of *B. aalborgi*, as well as *B. pilosicoli*, indicating that *B. aalborgi* could also cause symptomatic colitis.

## Introduction

Human intestinal spirochaetosis (HIS) was first reported by Harland and Lee in 1967.^(1)^ HIS is a disease of the colorectum caused by the two kinds of gram-negative bacteria *Brachyspira aalborgi* (*B. aalborgi*) and *Brachyspira pilosicoli* (*B. pilosicoli*).^(2)^ *B. pilosicoli* causes disease in humans and in various animals, however *B. aalborgi* induces colonic spirochaetosis only in humans.^(3,4)^

In clinical practice, HIS is often diagnosed upon pathological examination with a finding of so-called fringe formation.^(5)^ Definitive diagnosis requires the use of polymerase chain reaction, electron microscopy,^(6)^ imprint cytology,^(7)^ and detection of serum antibody titers to *B. aalborgi* and to *B. pilosicoli* with agglutination titers.^(8)^ We also isolated *B. aalborgi* and observed the genetic and immunological characteristics.^(9)^ All isolates also reacted with strain ATCC51139 and NCTC11492 strongly, but the reaction profiles attributed to our isolates were slightly different from those of ATCC51139 and NCTC11492. In 2017, we reported that a *Brachyspira*-like organism in an Akoya (pearl) oyster did not react with anti-*Treponema pallidum* (*T. pallidum*) serum but reacted with anti-*B. aalborgi* and anti-*B. pilosicoli* sera. This finding indicates that the antigenic features of *Brachyspira* sp. are different from those to *T. pallidum*.^(10)^

On the other hand, when we investigated the antibody to *B. hyodysenteriae* by dark field microscopy.^(11)^ the reaction was serotype-specific.^(12)^ We have been using this test in cases of human intestinal spirochetes, and the results indicate that the test is very useful and the reaction is serotype- or species-specific. We attempted to investigate the antibodies to the human brachyspiras and showed high titers in colitis patients with human intestinal spirochetes.

## Materials and Methods

*B. pilosicoli* ATCC51139, *Kyoto-C*, and *B. aalborgi* NCTC11492, *Yokosuka 24-d*, *B. ibaraki* (one lineage of *B. aalborgi*) were used for the agglutination test. The organisms were grown anaerobically on 4% sheep blood agar containing 400 µg/ml spectinomycin using a GasPak Anaerobic System (Mitsubishi Gas Chemical Co., Inc., Tokyo, Japan) as described previously.^(13)^ Dark field agglutination tests were carried out as described previously.^(11)^ *B. pilosicoli* was grown anaerobically for 7 days and *B. aalborgi* was grown anaerobically for 30 days. The cells grown on blood agar were harvested with physiological saline and after centrifugation, the precipitate was suspended in physiological saline. The cells were adjusted to MacFarland No.1. The sera collected from patients with diarrhea were diluted two-fold limiting dilution from 100 to 12,800. Into each well of the ceramic agglutination plate, an aliquot (0.05 µl) if the diluted serum pipetted and then 0.05 µl of the brachyspiral cell suspensions were dispensed into each well. After incubation for 60 min, the ceramic plates were left for 2 h. and then observed under the dark field microscope. Furthermore, the agglutination was observed again after 24 h and 48 h.

Informed consent was obtained from all subjects, and the experimental protocol was approved by the Ethics Committee of Tokyo Medical University Ibaraki Medical Center.

## Results

### Comparison of agglutinability between *B. pilosicoli* and *B. aalborgi*

**Fig. 1:**
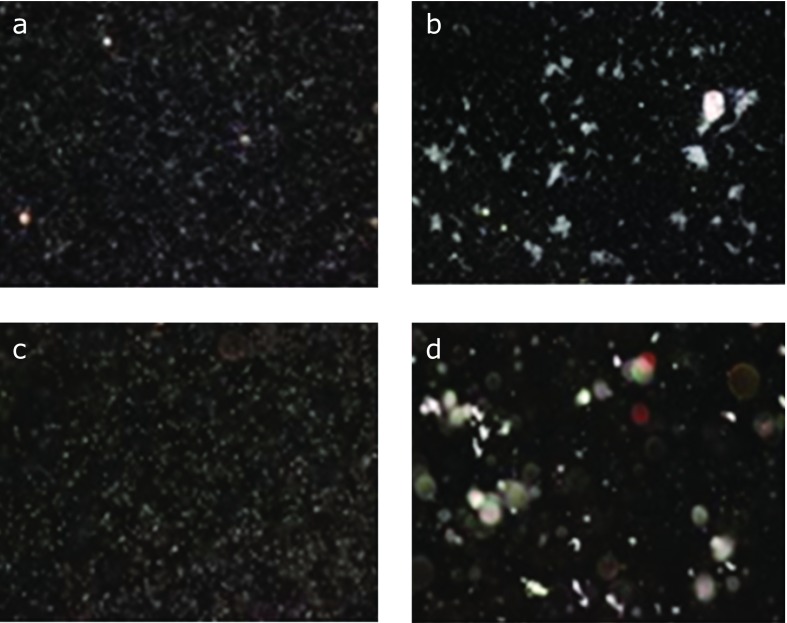
Comparison of agglutinability to between *B. pilosicoli* and *B. aalborgi*. (A, B) *B. pilosicoli*; (C, D) *B. aalborgi*. (A) and (C) were negative control (physiological saline was used instead of human serum). (B) and (D) were positive agglutination (human serum from a patient with colitis was used).

**Fig. 2:**
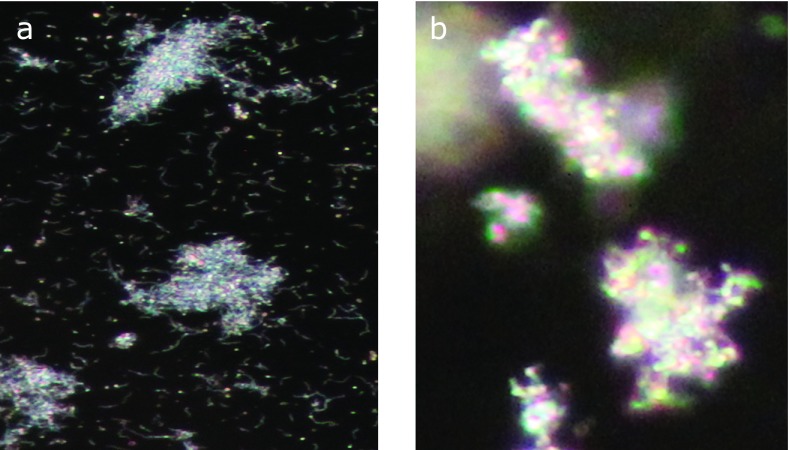
Comparison of intensity of the agglutinability between to *B. aalborgi* and to *B. pilosicoli*. (A) in agglutination with *B. pilosicoli*, free cells were observed and the agglutination was soft agglegate of the cells, while in agglutination with *B. aalborgi*, no free cells were observed and the agglutination rigid aggregate. As the results, the antibody titer to *B. aalborgi* was higher than that to *B. pilosicoli*.

Serum obtained from a patient with colitis was diluted at 1:100 and the agglutinability was compared between *B. pilosicoli* and *B. aalborgi* antigens adjusted to MacFarland No. 1. The results are shown in Fig. 1. Plates a and c were negative controls using physiological saline instead of human serum. Plates b and d showed positive agglutination using human serum from the colitis patient. In the agglutination reaction with *B. pilosicoli*, free cells were observed and the agglutination was a soft aggregate of the cells, while in agglutination with *B. aalborgi*, no free cells were observed and the agglutination formed a rigid cell aggregate (Fig. 2).

### Antibody titers on the agglutination reaction

The antibody titer to *B. pilosicoli* was higher than that to *B. aalborgi* in cases 1 and 2. By contrast, the antibody titer to *B. aalborgi* was higher than that to *B. pilosicoli* in case 3 (Table 1).

### Clinical feature of symptomatic patients with colitis who showed the high antibody titer of *B. pilosicoli*, or *B. aalborgi*

**Fig. 3:**
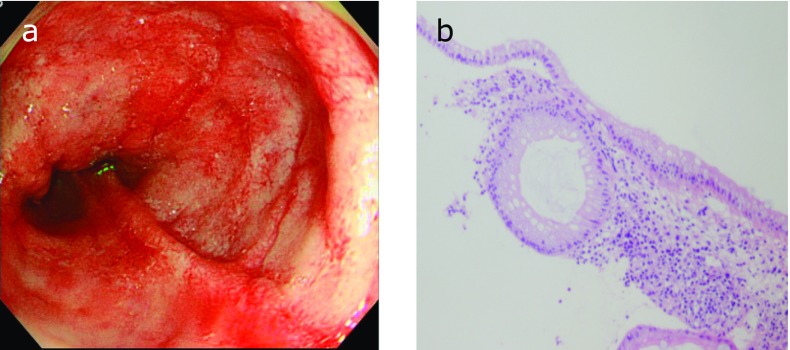
Case 1. Colonoscopy shows edematous mucosa with multiple erythematous spots in ascending colon (A) and histology of colonic biopsy specimens did not show fringe formation on the luminal side of colonic surface epithelium (hematoxylin-eosin stain) (B).

**Fig. 4:**
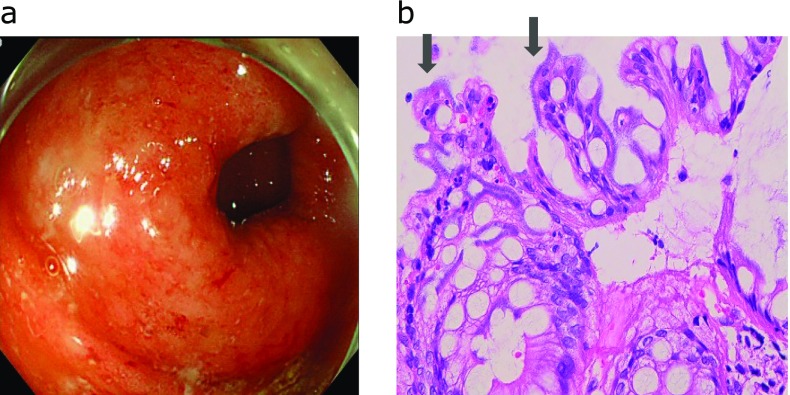
Case 2. Colonoscopy shows edematous mucosa with multiple erythematous spots in transverse colon (A) and histology of colonic biopsy specimens showed fringe formation on the luminal side of colonic surface epithelium (hematoxylin-eosin stain). Arrow showed human intestinal spirochetes (B).

The endoscopic and pathological findings of cases are shown in Fig. 3, 4, 5. The profiles of symptomatic patients with colitis with the high antibody titer for *B. pilosicoli*, or *B. aalborgi* are shown in Table 2. All three cases exhibited symptoms of colitis, including intense diarrhea, and colonoscopy revealed edematous mucosa with multiple erythematous spots in the colonic mucosa. These colonoscopic findings were mainly distributed in the ascending and transverse colon.

## Discussion

Previous studies have investigated the incidence of HIS in the colon; indicating its possible correlation with various diseases. HIS has been associated with carcinoma of the large intestine, adenomatous polyps, hemorrhoids with a metaplastic polyp, and ulcerative colitis.^(14)^ Another study has demonstrated that HIS was observed in cases of carcinoma or polyp of the colon and ulcerative colitis.^(15)^ We also have reported cases of HIS infection in ulcerative colitis patients undergoing long-term steroid therapy.^(16)^

We previously investigated the agglutination titers of five species of *Brachyspira* and showed high titers of *B. aalborgi* in the serum of patients.^(8)^ In our present study, we demonstrated high antibody titers of *B. pilosicoli*, or *B. aalborgi* in patients with human intestinal spirochaetosis-related colitis. In clinical practice, human intestinal spirochaetosis is often detected in cases of colonic polyps in the absence of symptoms, such as diarrhea. In our present cases, the patients exhibited the symptoms of colitis, including severe diarrhea, and HIS was detected in histopathological examination in two cases and all three cases exhibited high antibody titers for *B. pilosicoli*, or *B. aalborgi*. These results indicate that these patients could be diagnosed as colitis induced by human intestinal spirochetes.

The endoscopic findings of HIS-related colitis have been reported in several HIS case reports; edematous mucosa with multiple erythematous spots are observed in the ascending and in the proximal transverse colon and are not apparent in the distal colon or the rectum.^(5,17)^ Another previous report investigated the relationship between HIS infection and colonoscopic findings and the colonic distribution and showed that the colonoscopic findings in HIS patients exhibited either non-specific ulceration of the ileocecal valve or extensive areas of ischemic ulcers.^(18)^ The colonic distribution of 8 HIS-related cases of colitis has also been reported and 5 cases of the eight were throughout the entire colon, one case was right colon predominant, one case was rectal sparing and one case was right colon sparing.^(18)^ It has been also reported that colonoscopy revealed the edematous mucosa with multiple erythematous spots in the cecum and transverse colon and several ulcers were found in the transverse colon in HIS infectious colitis in an immunocompromised host.^(19)^ In the present cases, the colonoscopic findings exhibited edematous mucosa with multiple erythematous spots, which were similar to previous reports.

It has been suggested that *B. pilosicoli* infection causes clinical symptoms, such as abdominal pain and watery diarrhea.^(20)^ In the previous case report of *B. pilosicoli* infection, the patient had severe symptoms, such as acute onset abdominal pain and watery diarrhea.^(17)^ In another report from Japan, PCR analysis of *B. pilosicoli* and *B. aalborgi* were examined in asymptomatic HIS cases, and showed that more of the asymptomatic HIS patients were infected with *B. aalborgi*.^(21)^ These studies have suggested that patients with *B. pilosicoli* infection exhibit more severe symptoms, such as abdominal pain and watery diarrhea, than patients with *B. aalborgi* infection. In the present cases, severe symptoms, such as abdominal pain and diarrhea, were observed in cases with high antibody titers for both *B. pilosicoli* and *B. aalborgi*, indicating that *B. aalborgi* could also cause symptomatic colitis.

The recent investigation has demonstrated the probable association between proton pump inhibitor (PPI) use and alternation of gut microbiota, indicating that PPIs may predispose the enteric infection.^(22)^ From these valuable reported results, the effect of PPI use on *B. pilosicoli* and *B. aalborgi* infection should be investigated in the next study.

The limitation of this study is that the agglutination titers of *B. pilosicoli* and *B. aalborgi* were examined in only three cases of colitis patients with human intestinal spirochetes. Therefore, accumulation of human intestinal spirochetes cases examined with agglutination titers of *B. pilosicoli* and *B. aalborgi* is desirable.

In conclusion, in cases of HIS infection, the differential diagnosis of *B. pilosicoli* and *B. aalborgi* is clinically meaningful because it has been suggested that the clinical feature of these two kinds of infection are different. Severe symptoms, such as abdominal pain and diarrhea, were observed in cases with high antibody titer of *B. pilosicoli* and *B. aalborgi*, indicating that *B. aalborgi* could also cause symptomatic colitis.

## Figures and Tables

**Fig. 5:**
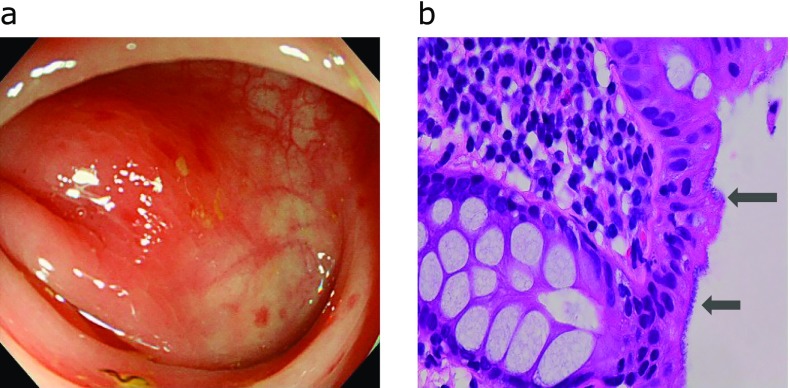
Colonoscopy shows edematous mucosa with multiple erythematous spots in transverse colon (A) and histology of colonic biopsy specimens showed fringe formation on the luminal side of colonic surface epithelium (hematoxylin-eosin stain). Arrow showed human intestinal spirochetes (B).

**Table 1:** Diagnosis of patients with colitis by dark field agglutination test

	**Agglutination titers to**		
Patients	Specimens	*B. p* (*Kyoto-C*)	*B. a* (*B. ibaraki*)
Case 1		3,200 (after 24 h)	200> (after 24 h)
Case 2		6,400 (after 24 h)	200> (after 24 h)
	
	Specimens	*B. p* (ATCC51139)	*B. a* (*B. ibaraki*)
	
Case 3		80 (after 24 h)	640 (after 24 h)

Agglutination titers were investigated under dark field microscopy. *B. p*, *Brachyspira pilosicoli*; *B. a*, *Brachyspira aalborgi*.

**Table 2:** Clinical and endoscopical findings of three cases

Case	Age/Gender	Underlying disease	Clinical symptom	Endoscopic findings	Colonic distribution of HIS	Severity of histopathological inflammation (microscopic findings)	Dignosis of HIS
1	31/male	Ulcerative colitis	Watery diarrhea Abdominal pain	Edematous mucosa with multiple erythematous spots	A/C, T/C	Moderate	*B. p*
2	53/male	None	Watery diarrhea Abdominal pain	Edematous mucosa with multiple erythematous spots	T/C	Moderate	*B. p*
3	58/male	None	Watery diarrhea Abdominal pain	Edematous mucosa with multiple erythematous spots	A/C, T/C, S/C	Mild	*B. a*

*B. p*, *Brachyspira pilosicoli*; *B. a*, *Brachyspira aalborgi*.
